# Evaluation of Clinical Preventive Management Provided by Primary Healthcare Physicians Against Brucellosis in Saudi Arabia

**DOI:** 10.7759/cureus.52841

**Published:** 2024-01-24

**Authors:** Hani S Almugti, Noof M Shaheen, Lina Al Anazi, Ahmed A Al zahrani, Saeed A Al Ghamdi, Maram F Al Mehmadi, Turkyah J Al Bogami, Hussain Al Qattan, Mishal M Al Motairi, Jumanah A Al Taha, Mashael Al Qahtani, Mousa Z Al Mutairi, Afit Al Sharari, Munirah Al Ajlan, Hameed J Al Enazi

**Affiliations:** 1 Primary Health Care, King Abdullah International Medical Research Center, King Saud bin Abdulaziz University for Health Sciences, Ministry of National Guard Health Affairs, Jeddah, SAU; 2 Research, Dr. Salam Jibrel Medical Center, Manama, BHR; 3 Emergency Medicine, Ministry of Health, Riyadh, SAU; 4 College of Medicine, Al-Baha University, Al-Baha, SAU; 5 General Practice, Almikhwah Primary Heathcare Center, Mulaija, SAU; 6 Microbiology, Jeddah Regional Lab, Jeddah, SAU; 7 Biochemistry, Faculty of Science, King Abdulaziz University, Makkah, SAU; 8 Emergency Medicine, Prince Sultan Hospital, Mulaija, SAU; 9 Renal Dialysis Unit, Security Forces Hospital, Riyadh, SAU; 10 Internal Medicine, Al-Omran General Hospital, Al-Ahsa, SAU; 11 Laboratory, Al Muzahimiyah General Hospital, Riyadh, SAU; 12 Laboratory, Forensic Medical Services Center, Al Madinah, SAU; 13 College of Medicine, Ibn Sina National College for Medical Studies, Jeddah, SAU; 14 Pharmaceutical Care Services, Ad-Diriyah Hospital, Ministry of Health, Riyadh, SAU; 15 Nursing, Security Forces Hospital, Riyadh, SAU

**Keywords:** saudi arabia, preventive medicine, primary care, reporting, brucellosis

## Abstract

Background: Brucellosis is among the most common zoonotic bacterial infections, leading to major public health consequences in endemic areas such as Saudi Arabia. Primary healthcare is crucial in controlling brucellosis, as it serves as the frontline for disease prevention, early detection, and appropriate management. However, enhancing the contribution of primary healthcare to the entire brucellosis notification process is necessary to minimize the underreporting and inadequate data collection, which hinders the implementation of effective control measures.

Objective: The objective of the study is to assess primary care physicians' knowledge and practice of clinical preventive management in Saudi Arabia regarding brucellosis using an adapted assessment tool featuring a semi-structured questionnaire.

Subjects and methods: The current study's design is a cross-sectional study based on a questionnaire. Three hundred and seventy-three primary healthcare physicians in Saudi Arabia were chosen for self-administered online standardized questionnaires.

Results: One-third of the participants answered all the knowledge assessment questions correctly. Most participants had more than 10 years of professional experience and were 40 or older. In response to the practice assessment questions, 210 physicians stated that they had encountered at least one case of brucellosis, and two-thirds had no compliance with the notification process of their cases.

Conclusion: The limited knowledge and improper practice of primary care physicians regarding human brucellosis are possible underlying reasons for the underdiagnosis and underreporting of brucellosis patients at primary health care clinics in Saudi Arabia. Most research indicates that implementing specific educational programs to improve knowledge is necessary for primary healthcare workers. Furthermore, enhancing the community interaction between healthcare centers and the community facilitates effective control measures against brucellosis.

## Introduction

Brucellosis is among the most common zoonotic bacterial infections, leading to major public health consequences in endemic areas such as Saudi Arabia [1]. Each year, there are more than 500,000 new cases of reported human brucellosis worldwide [2]. In Saudi Arabia, 37,477 reported cases of human brucellosis occurred between 2004 and 2012 [1]. According to a Saudi study, the incidence rate significantly dropped from 22.9 in 2004 to 12.5 in 2012 [3]. However, based on a recent report [4] from the Saudi Ministry of Health, the number of brucellosis cases that health professionals reported decreased by 50% between 2017 and 2021.

Both humans and livestock are susceptible to brucellosis, which typically spreads to humans by direct contact with infectious materials, such as afterbirth, or indirectly through the consumption of animal products [1]. Because of this transmission mode, brucellosis is one of the common zoonosis illnesses that exemplifies one health concept. In Saudi Arabia, a higher number of livestock comes from outside due to traditional demands and religious requirements related to the Hajj and Umrah. Globally, there are variations in the prevalence of brucellosis in livestock. Still, in Saudi Arabia, the prevalence is estimated to be 17.4%, reaching up to 26.1% in the Al-Qassim and Riyadh regions [5].

Reporting is essential for effective brucellosis surveillance; therefore, healthcare systems are strongly encouraged to practice reporting. The underreporting of brucellosis cases is a concern in the medical literature [6]. Unspecified clinical symptoms of this disease are considered an attributing factor for underreporting. Moreover, levels of knowledge and practice among primary care physicians are additional contributing factors to the under-diagnosing and under-reporting of brucellosis cases, according to a Saudi study [7]. Although brucellosis has been studied in Saudi Arabia from different perspectives, this research highlighted the challenges against reporting brucellosis from the perspective of primary healthcare physicians in several Saudi health sectors.

The current study aimed to improve the compliance of primary healthcare physicians to the reporting practice and other clinical preventive medicine practices against brucellosis. The objective was to assess primary care physicians' knowledge and practice in Saudi Arabia regarding the clinical preventive measures of brucellosis using an adapted assessment tool consisting of a semi-structured questionnaire.

## Materials and methods

Study area/setting and subjects

The study was carried out in primary health centers in Saudi Arabia. Primary healthcare centers are the first line of interaction between the patient and the healthcare system and the frontline for reporting notifiable diseases. The study subjects were the primary healthcare physicians who currently work in Saudi primary healthcare centers of the following health institutes (Ministry of Health, Ministry of National Guard, health services of Ministry of Defence, and health services of Ministry of Interior). We excluded the interns and students from the medical college from participation in the present study.

Study design and sampling

The current study design is cross-sectional, and through snowballing (non-probability sampling), the study included primary healthcare physicians who met the inclusion and exclusion criteria using a self-administered online questionnaire.

Using Epi Info statistical software, version 7.2, the sample size of 362 primary healthcare physicians was calculated at a confidence level of 95% and a 5% margin of error. This sample size was estimated at 6,107 [8], representing the total number of primary healthcare physicians in Saudi Arabia who met the inclusion and exclusion criteria.

The electronic questionnaire was utilized to contact participants; it was shared in November 2023 on Twitter, Facebook, and WhatsApp, three of the most popular social media platforms among Saudis.

Data collection methods, instrument used, and measurements

Variables

The primary healthcare physicians' knowledge and practice assessment outcomes about brucellosis clinical preventive management were the dependent variables. In contrast, the independent variables included age, gender, nationality, profession (Job title), length of experience, and type of primary health care center (Ministry of Health, Ministry of National Guard, health services of Ministry of Defence, and Ministry of Interior).

Operation Definition

According to the scientific guidelines, physicians' knowledge was measured by correctly answering the clinical questions related to Brucellosis regarding clinical description, laboratory criteria for diagnosis, reporting, mode of transmission, and drug prescription details (name of drug-dose-frequency-duration).

The physicians' practice was measured by how correctly, during the past two years and according to the scientific guidelines, the physicians managed their brucellosis cases, including drug prescription details (name of drug-dose-frequency-duration), health education provided, clinical counseling activities, and the adherence to the reporting practice for such cases.

Questionnaire

Five primary healthcare consultants participated in a face-to-face validation testing of the questionnaire. The reliability of the questionnaire was tested by distributing 10 questionnaires among primary care physicians at King Abdulaziz Medical City in Jeddah. The reliability percentage is 84.1 %, indicating very good reliability. The questionnaire was self-administered and consisted of three sections; the first section included age, gender, nationality, profession (Job title), length of experience, and type of primary health care center (Ministry of Health, Ministry of National Guard, and Health Services of Ministry of Defence). Six closed-ended questions were used in the second section to assess the participants' knowledge about Brucellosis. The last section of the questionnaire comprised practice-based assessment questions, and responses were evaluated following scientific guidelines. 

Data management and analysis plan

Data entry and analysis were performed using IBM SPSS Statistics for Windows, Version 20 (Released 2011; IBM Corp., Armonk, New York, United States). The data entry and coding stages were carried out to improve the data quality. Data is displayed using percentages, frequencies, means, and standard deviations for qualitative variables. The Chi-square test was used between participants' answers and other variables to record the statistical significance.

## Results

Demographic and occupational information

**Figure 1:**
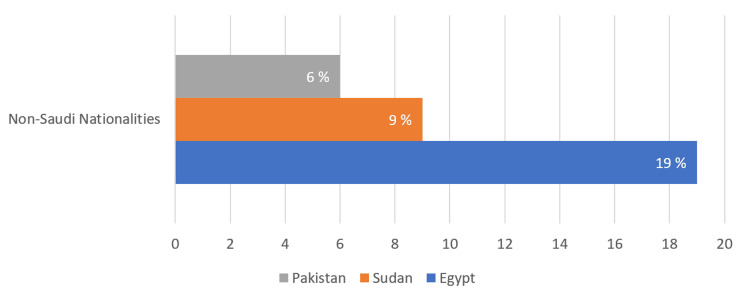
The percentages of non-Saudi primary health physicians from different nationalities

The current study included 373 primary healthcare physicians who answered the online questionnaire. Table 1 shows that two-thirds of the participants were from the age group of more than 30 years and had work experience of more than five years. Of the invited physicians, 62.5% were Saudis, 11 % were working for the health services of the Ministry of Interior, and 19 % were from Egypt (Figure 1).

**Table 1:** Demographic and occupational characteristics of participants (n=373)

Demographic characteristics	Frequency (n)	Percent (%)
Age category		
20 - 30 years	99	26.5
31 - 40 years	163	43.7
41 - 50 years	111	29.8
Gender		
Male	169	45.3
Female	204	54.7
Nationality		
Saudi	233	62.5
Non-Saudi	140	37.5
Health Sectors		
Ministry of Health	122	32.7
Ministry of National Guard Health Affairs	111	29.8
Health services of the Ministry of Defense	96	25.7
Health services of the Ministry of Interior	44	11.8
Length of Experience		
Less than five years	93	25
From five to ten years	70	19
More than ten years	210	56

Participants' knowledge assessment regarding clinical preventive management of brucellosis

As shown in Table 2, slightly two-thirds of participants answered correctly to the knowledge assessment questions regarding the laboratory diagnosis of Brucellosis and human-to-human transmission risk. However, one-third of them are unaware that brucellosis can manifest clinically as acute or insidious, while another third stated wrongly that the type of brucellosis notification is weekly.

**Table 2:** Participants' knowledge about clinical preventive management of brucellosis (n=373)

Questions of Knowledge Assessment	Number of Correct Answers (%)	Number of Wrong Answers (%)
What is the clinical description of Brucellosis?	134 (36 %)	239 (64 %)
Which of the following laboratory criteria for diagnosis is considered definitive?	280 (75 %)	93 (25 %)
Type of notification (immediate – Weekly – Monthly)?	140 (37.5 %)	233 (62.5 %)
Human to human transmission risk of brucellosis	239 (64 %)	134 (36 %)

Assessment of participants' clinical practice with brucellosis cases

**Figure 2:**
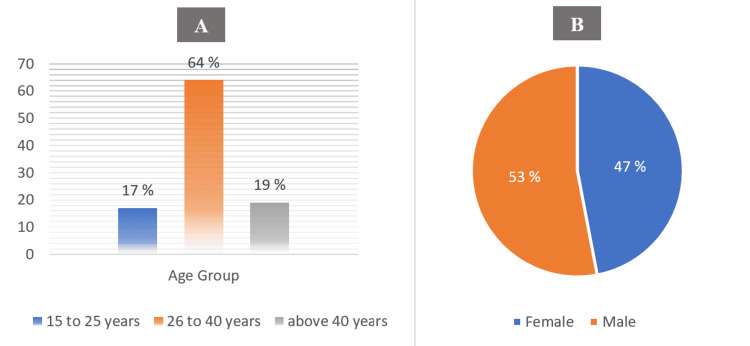
Percentage of different age groups (A) and percentage of gender (B) of the brucellosis cases (n=210)

In response to the practice assessment questions, 210 physicians have experience managing brucellosis cases in the previous two years. About two-thirds of these cases were from the age group of 26 to 40 years, with approximately 1:1 sex ratio (Figure 2).

Table 3 illustrates that at least 50 percent of participants managed their cases in best clinical practice according to the scientific guidelines. However, this table shows that almost half of them prescribed the incorrect treatment course, including the name of the drug regimen, frequency, and duration. Furthermore, two-thirds of them needed to comply with the reporting practice of their cases.

**Table 3:** Participants' clinical practice with the brucellosis cases (n=210)

Questions of Practice Assessment	Yes (%)	No (%)
Request a definitive method of diagnosis	175 (83 %)	35 (17 %)
Prescribe a correct treatment course.	111 (53 %)	93 (47%)
Filing the form of notification	76 (36 %)	134 (64 %)
Providing clinical counseling sessions?	169 (80 %)	41 (20 %)

Relation between the assessment of knowledge of participants and their demographic and occupational characteristics

Responding to the last question, participating physicians were asked about their knowledge of community services against Brucellosis provided by their primary care centers; their responses are indicated in Table 4. Participants who confirmed their awareness about community services were from National Guard Health Affairs, while the other participants expressed uncertainty about community services. In testing the association between knowledge and demographic/occupational characteristics, Table 4 demonstrates that out of the participants, one-third answered all of the questions correctly. Among those participants, almost half had more than ten years of professional experience and were 40 or older. Furthermore, Table 4 shows a higher rate of correct answers among physicians affiliated with the Ministry of Health and Health Services of the Ministry of Defense compared with physicians from other sectors.

**Table 4:** Relation between participants' knowledge assessment and their demographic characteristics (n=373) (*) Statistically significant at p<0.05 (Chi-square test)

Demographic characteristics	Questions of Knowledge Assessment		
	Wrongly Answering any question (n)	Correctly answering all Questions (n)	p-value (Chi-square test)
Total	248 (67 %)	125 (33 %)	
Age category			
20 - 30 years	60	39	0.08
31 - 40 years	132	31	
41 - 50 years	56	55	
Gender			
Male	109	60	0.45
Female	139	65	
Nationality			
Saudi	161	72	0.17
Non-Saudi	87	53	
Health Sectors			
Ministry of Health	77	45	0.001*
Ministry of National Guard Health Affairs	84	27	
Health Services of the Ministry of Defense	29	41	
Health Services of the Ministry of Interior	58	12	
Length of Experience			
Less than five years	54	39	0.003*
From five to ten years	58	12	
More than ten years	136	74	

## Discussion

Human brucellosis is a major global public health concern. Primary healthcare physicians play a crucial role in the early detection, notification, diagnosis, and treatment of brucellosis infections. The objective of the study was to assess primary care physicians' knowledge and practice in Saudi Arabia regarding the clinical preventive management of brucellosis; compared with the previous Saudi study [7], the present study was more generalized and targeted primary healthcare physicians working in the main health sectors in Saudi Arabia. Furthermore, most of the participants in this study were from the age group of more than 30 years and had work experience of more than five years. This implies that they have long clinical experience and encountered more brucellosis cases, which enhances the outcome of assessing their knowledge and adherence to the clinically recommended preventive practices against brucellosis.

The percentage of brucellosis cases among males and females in this study was almost equal. Our findings confirm the hypothesis that the disease affects both genders. However, a previous Iranian study [9] found that the incidence rate was higher in women, and another study from Saudi Arabia reported that it was higher in men [10]. The higher incidence rate among men is attributed to the cultural or lifestyle factors in Saudi Arabia, where males are more likely than females to consume raw milk, and they often spend several days camping in rural areas, enjoy drinking the fresh camel milk that is offered by local livestock farmers [11,12].

Previous studies [13,14] have addressed underlying causes for underreporting brucellosis cases and identified several contributing factors, such as nonspecific manifestation of the disease and limited medical knowledge about the clinical symptoms, diagnosis process, and disease transmission mechanism among healthcare workers. In the present study, only one-third of participants answered all of the questions correctly; the findings of the present study are consistent with the results of the previous Saudi study [7] and support the need for education programs among physicians to overcome the challenges of underestimation and underreporting of brucellosis cases.

Following the Primary Healthcare Accreditation Standards of the Central Board of Accreditation for Healthcare Institutions (CBAHI) [15], the primary healthcare center is responsible for health promotion and education to the community at a large scale. Despite the endemicity of brucellosis in Saudi Arabia, the current study found that most physicians expressed uncertainty about the community services provided by their healthcare centers to control Brucellosis. The low collaboration among healthcare centers necessitates the attention of the healthcare system to provide the needed community health education. The continuous monitoring of relevant health authorities is essential in ensuring that these centers comply with quality standards.

Although the current study includes primary healthcare physicians from various healthcare sectors in Saudi Arabia, it is important to acknowledge its numerous limitations. One of them is that this is a cross-sectional study, and cause and effect relationship cannot be established. Furthermore, the inclusion of primary care physicians without considering other healthcare professionals, such as nurses and laboratory teams, might provide a general assessment of how to manage brucellosis cases in primary healthcare centers. Furthermore, this study was approached through self-reported responses to questions about clinical practices. However, incorporating a review of patient records and treatment protocols would have yielded more comprehensive findings, enhancing the overall assessment of current medical practices.

## Conclusions

The limited knowledge and improper practice of primary care physicians regarding human brucellosis are possible underlying reasons for the underdiagnosis and underreporting of brucellosis patients at primary healthcare clinics in Saudi Arabia. Most research indicates that implementing specific educational programs to improve knowledge is necessary for primary healthcare workers. Furthermore, quality-focused institutions advise that in order to control brucellosis, community interaction between healthcare centers and the community needs to be activated in order to disseminate health information related to brucellosis preventive measures.

## References

[1] Al Anazi M, AlFayyad I, AlOtaibi R, Abu-Shaheen A (2019). Epidemiology of brucellosis in Saudi Arabia. Saudi Med J.

[2] (2023). CDC Yellow Book 2024. https://wwwnc.cdc.gov/travel/yellowbook/2024/infections-diseases/brucellosis.

[3] Aloufi AD, Memish ZA, Assiri AM, McNabb SJ (2016). Trends of reported human cases of brucellosis, Kingdom of Saudi Arabia, 2004-2012. J Epidemiol Glob Health.

[4] (2023). MOH Statistical Yearbook 2021. Yearbook.

[5] Al-Hakami AM, Alqahtani AJ, Moosa RA (2019). Seroprevalence of brucellosis among exposed agro-pastoral communities in southern Saudi Arabia. Asian Pac J Trop Med.

[6] Mohamed AA, Chehab MA, Al-Dahshan A, Al-Romaihi HE, Farag EA (2019). An evaluation of the national brucellosis surveillance system in Qatar, 2018. Cureus.

[7] Alrajhi MM (2020). Primary health care physicians' knowledge on brucellosis, its prevention, diagnosis, and management--a cross section survey in Prince Sultan Military Medical City, Riyadh, Saudi Arabia. J Evol Med Dent Sci.

[8] Al-Khaldi YM, Al-Ghamdi EA, Al-Mogbil TI, Al-Khashan HI (2017). Family medicine practice in Saudi Arabia: the current situation and Proposed Strategic Directions Plan 2020. J Family Community Med.

[9] Nematollahi S, Ayubi E, Karami M (2017). Epidemiological characteristics of human brucellosis in Hamadan Province during 2009-2015: results from the National Notifiable Diseases Surveillance System. Int J Infect Dis.

[10] Memish Z (2001). Brucellosis control in Saudi Arabia: prospects and challenges. J Chemother.

[11] Alkahtani AM, Assiry MM, Chandramoorthy HC, Al-Hakami AM, Hamid ME (2020). Sero-prevalence and risk factors of brucellosis among suspected febrile patients attending a referral hospital in southern Saudi Arabia (2014-2018). BMC Infect Dis.

[12] Benjamin B, Annobil SH (1992). Childhood brucellosis in southwestern Saudi Arabia: a 5-year experience. J Trop Pediatr.

[13] Lai S, Chen Q, Li Z (2021). Human brucellosis: an ongoing global health challenge. China CDC Wkly.

[14] Dean AS, Crump L, Greter H, Schelling E, Zinsstag J (2012). Global burden of human brucellosis: a systematic review of disease frequency. PLoS Negl Trop Dis.

[15] (2023). Saudi Central Board for Accreditation of Health Institutions : Primary Healthcare Accreditation Standards. https://portal.cbahi.gov.sa/english/cbahi-standards.

